# The Effect of Baby Schema in Cats on Length of Stay in an Irish Animal Shelter

**DOI:** 10.3390/ani12111461

**Published:** 2022-06-04

**Authors:** Sam Jack, Grace A. Carroll

**Affiliations:** Animal Behaviour Centre, School of Psychology, Queen’s University Belfast, David Keir Building, 18-30 Malone Road, Belfast BT9 5BN, UK; g.carroll@qub.ac.uk

**Keywords:** baby schema, infant features, cats, animal welfare, animal shelter, companion animals, Length of stay, animal adoption

## Abstract

**Simple Summary:**

Several factors may influence cats’ length of stay (LoS) within the shelter environment. The aim of this study was to investigate the potential influence that ‘cuteness’ has on cats’ length of stay (LoS) in an animal shelter. Other factors such as paired-homing requirements, adoption profile (advertisements to encourage the adoption of a specific animal) wording, coat colour, sex and age were also explored. ‘Cuteness’ was measured in two ways; 1. Facial characteristics which characterise human infants, such as big eyes and a round head, were measured from images of cats to create an objective cuteness score. 2. Subjective cuteness scores were developed by participants rating the ‘cuteness’ of the cat images from ‘1-Not very cute’ to ‘5-Extremely cute’ through two online surveys; survey 1 investigated the cats’ social nature as well as cuteness while the second only looked at cuteness. The subjective cuteness scores were used to validate the objective cuteness scores. The analysis found that subjective cuteness influenced cats’ length of stay during the analysis of the first survey with no effect of other variables such as adoption profile language, homing requirements, coat colour, sex and age. Analysis of the data from the second survey found that none of the variables influenced the cats’ LoS. A novel objective measurement for cuteness in cats was developed which reflects the shape of the eyes and is associated with subjective cuteness scores. This research looks to understand factors that influence LoS to help identify potential methods to reduce time spent in shelters and improve the welfare of cats within these environments.

**Abstract:**

Several factors may influence cats’ length of stay (LoS) within the shelter environment. The aim of this study was to investigate the potential influence that baby schema (characteristic facial features of infants, related to perceived ‘cuteness’) has on cats’ length of stay (LoS) in an animal shelter. Other factors such as paired-homing requirements, adoption profile (adverts to encourage the adoption of a specific animal) wording, coat colour, sex and age were also explored. Objective ‘cuteness’ scores were created by measuring specific facial features of 165 shelter cats. Several proportions of the cats’ faces were explored to identify the best objective measure of cuteness, including elements which have been found to associate with cuteness in cats and humans and new exploratory eye measurements. Subjective cuteness scores were developed by participants rating the ‘cuteness’ of the cat from ‘1-Not very cute’ to ‘5-Extremely cute’ through two online surveys; survey 1 investigated the cats’ social nature as well as cuteness while the second only looked at cuteness. The subjective cuteness scores were used to validate the objective cuteness scores. The analysis found that subjective cuteness in survey 1 was the only variable of influence on LoS. A novel objective cuteness measurement was developed which reflects the eye shape associated with subjective cuteness scores. The current study found that cuteness may not be as influential on cats’ LoS in shelters as hypothesised. This research looks to understand factors which influence LoS to help identify potential methods to reduce time spent in shelters and improve the welfare of cats within these environments.

## 1. Introduction

Cats do not cope well with shelter life. The longer they are housed within these environments, the greater the chance they will be euthanised, contract an infection or develop negative behavioural traits [1,2]. There are thousands of unwanted cats housed throughout several animal charities in the United Kingdom and Ireland. In 2019, 41,000 cats were rehomed by the charity Cats Protection in the UK alone [3]. Previous studies have explored multiple factors affecting the speed of cat adoption, or, Length of Stay (LoS), in shelters, including phenotypic and behavioural characteristics such as coat colour, length and markings, temperament, facial expressions and vocalisations [4,5,6,7,8]. Several studies have found that males are adopted the quickest, particularly young, lighter coloured males, whilst darker coloured females tend to take the longest to rehome [4,7,8]. In addition, there is evidence to suggest that information contained within adoption profiles may affect LoS. Adoption profiles are adverts created by shelters to encourage the adoption of a specific animal, and usually contain an image of the adoptee along with a blurb regarding the social nature or temperament of the animal. Housing requirements for each animal are often specified, such as ‘requires access to outside’, ‘no other pets’ and ‘only to be housed with children over 12 years old’. In a recent study, Rix et al. [9] found that while cat name type (for example, a ‘human’ name compared to food or plant-themed names) did not affect LoS, cats with a profile that was written in the third person (*“Tiger is a handsome chap…”)* as opposed to the first-person (*“Hi my name is Bear, I am a sweet boy…”)* were adopted more quickly. It is possible that other profile characteristics may affect LoS. For example, previous research suggests that temperament is one key reason for selecting a particular animal [5,6,10] and adoption profiles can give potential adopters an indication of the animals’ disposition. Another factor that could potentially affect LoS is paired-homing requirements. Cats may be relinquished as pairs or if the shelter is a grouped housing system, some may bond. In such cases, shelters sometimes require an adopter who would accept both individuals. This may be harder to accommodate as adopters may be discouraged by the implications of adopting two cats rather than one, such as an increase in both financial expenses (adoption fees, veterinary bills, increase in food bills, etc.) and responsibility. 

Another key trait that may affect LoS but has yet to be assessed, is that of baby schema. Originally termed ‘*Kindchenschema*’, the baby schema is the umbrella term for specific features, mostly facial, which characterise infants: large head with a protruding forehead, big low-lying eyes, bulging cheeks and a small nose and mouth [11]. These features play a major role in human infant development and survival due to their influence on parental attachments and they affect how infants are perceived and treated [12,13,14,15]. Baby schemas are an important factor in attracting and inciting the motivation of caregivers [16], with the parents of ‘cute’ babies shown to provide them with more affection and attention than those that are regarded as less ‘cute’ [12,13]. Cuteness and what individuals find attractive are highly subjective. However, infant features have been linked to increases in perceived cuteness in humans, non-animate objects, and animals [17,18,19,20,21]. Objective measures of baby schema have been developed previously through facial parameter measurements and manipulations. These objective measurements were found to be associated with subjective cuteness scores, which were established by participants rating the ‘cuteness’ of a set of infant-related images [16,18,22,23]. Glocker et al. [16] developed a protocol that captures baby schema with six facial parameters and then used these to manipulate images of human infants to express high and low levels of baby schema. The high baby schema images were rated as ‘cuter’ than the low baby schema images when assessed by a panel of participants. Archer and Monton [18] did a comparative study of images of adult and infant humans, as well as cats, dogs and teddy bears, which expressed high and low levels of baby schema. This study found that the images with a larger forehead to face length ratio were the ones that expressed high levels of baby schema and were found to be the most attractive. Infant cuteness plays a role in adoption preference, with cuteness being the most influential factor in adoption decisions for women and second in importance for men [24]. As cuteness plays a key role in human adoption and acquiring attention from caregivers, it may also be an important factor in the adoption of cats from shelters. For example, this may explain why kittens are adopted quicker than older animals, as pet owners have been found to have a strong preference for infant features in animals [18]. The effect that cuteness has on adoption choice in human infants could be the result of the ‘what is beautiful is good’ stereotype; the bias that physical attractiveness equates to positive personality traits [24,25]. Human infant attractiveness influences how the child is perceived, with cuteness being associated with good and positive behaviour, whilst those perceived to be less attractive often being assumed to have more difficult and negative behavioural traits [26]. In animal adoption, the way an animal looks may influence how potential adopters perceive the animal’s social nature or temperament and may result in less attractive animals being viewed as having a more negative social nature.

Facial expressions have an influence on the adoption rate for dogs, with a preference for those who have the ability to raise their eyebrows, thus making their eyes appear larger; a significant paedomorphic attribute [27] For cats in shelters, there is no influence of facial expression on adoption success [5]. However, the effect of Baby Schema in adult cats on LoS in animal shelters has yet to be explored. Only Archer and Monton [18], Little [23] and Borgi et al. [22] have explored the influence of baby schema in cats, but they used manipulated professional versions of the same animal image or used images that varied widely in their possession of infant features. Therefore, it is likely that a more species-specific measure may be needed to distinguish between high and low baby schema in shelter cats in terms of being precise enough to detect the subtle differences in the baby schema.

Using retrospective data from an animal shelter, the primary aim of this research was to determine the effect of baby schema in adult cats on Length of Stay (LoS). Several objective measures were explored using previously developed methods from Glocker et al. [16] and Archer and Monton [18]. Novel facial measuring procedures were also investigated as previous studies used methods that had been developed originally for use on human infants. LoS was further explored by assessing the effect that positive and negative wording in adoption profiles, paired-homing restrictions, and other cat characteristics such as sex, age, reason for relinquishment and coat colour had on length of stay.

**Hypothesis** **1.** *‘Cute’ cats, as determined by subjective and objective measures, will have a shorter LoS than less ‘cute’ cats.*

**Hypothesis** **2.** *Cats with ‘positive’ adoption profiles will have shorter LoS compared to cats with ’negative’ adoption profiles.*

**Hypothesis** **3.** *Cats with paired-homing requirements will take longer to adopt than those without paired-homing requirements.*

**Hypothesis** **4.** *Cats rated as ‘cuter’ will be perceived as having a positive nature compared to those with lower cuteness ratings.*

## 2. Materials and Methods 

### 2.1. Ethical Statement 

Ethical approval was obtained from the Queens University Belfast EPS Faculty Ethics Committee (EPS 20_50). All participants gave informed consent. One Irish animal shelter consented to provide their data.

### 2.2. Study Population and Inclusion/Exclusion Criteria 

The intake and adoption details of cats housed within the ISPCA centre in Longford, Ireland from the period of January 2015 to September 2019, were collected. When placed within the shelter, each cat has its name, sex, an estimation of age, and its intake date documented on a Microsoft Excel spreadsheet. The cat’s relinquishment status is also recorded as one of the following: ‘unable to cope’; ‘abandoned’; ‘stray’; ‘sick/injured’; ‘temporary admission’; ‘neglect’; ‘born at the shelter’; ‘hoarding/breeding establishment’; ‘unsuitable accommodation’; ‘rescued from laboratory’. No details of the cats’ phenotypic features, such as coat colour or length are recorded using this system. When adopted, the cat’s details, along with the adoption date, are marked on a separate spreadsheet. A total of 1281 intake and adoption records were available, with the average length of stay for an adult cat (over the age of 1 year) as 80 days. The following inclusion and exclusion criteria were applied; only cats over the age of one year old were included in the study. This is because kittens are typically relinquished at lower rates and adopted quicker than adult cats [9,28]. Therefore, it is of greatest importance to understand adoption decisions in terms of adult cats. In addition, the exclusion of cats under one year of age allowed us to look specifically at the effects of paedomorphosis, i.e., the retention of juvenile traits into adulthood [29], on adoption decisions. To be included in the study, the cats had to have both intake and adoption details, along with an adoption profile that included details of rehoming requirements, and a suitable photo. Suitable images had to have a minimum of medium resolution (300 ppi-pixels per inch minimum, the industry standard for printing) with the cats’ faces clearly visible and facing forward. Only cats with a neutral facial expression (mouth closed, ears facing forward, eyes open) and those which had no visible injuries or deformities to the face, such as missing eyes, ears, or wounds, were included. Only the cat’s face was included in the survey to ensure there was no influence from other visual stimuli, such as background or body. Due to missing information in adoption profiles, of the 1281 records, only 77 cats met the criteria for an assessment of the effects of adoption profile, paired-housing requirements, and baby schema on LoS. Therefore, there were 77 cats included in the first online survey. In a second online survey, adoption profile and housing restrictions were excluded, which allowed an additional 88 cats to be included. A total of 165 cats had adoption details and images of sufficient quality to be included in this analysis.

### 2.3. Data Preparation

Several new variables were created based on the 165 cat images and the 77 adoption profiles. These are described below.

#### 2.3.1. Adoption Profiles 

Adoption profiles were categorised as either ‘positive’ or ‘negative’. Those that were designated as ‘positive’ used descriptions such as ‘friendly’ and ‘affectionate’ to describe the cat, whilst those assigned to the ‘negative’ group used language and expressions such as ‘quiet’, ‘reserved’ or ‘affectionate on own terms. The adoption profiles were also gleaned for information regarding the cats’ paired-homing requirements; cats were assigned to ‘paired’ if there was a requirement for the cat to be rehomed along with another specified cat. Any other cat was classified as ‘single’.

#### 2.3.2. Coat Colour 

The Cat Identification Coat Guide was used to identify and place the cats into coat colouring groups [30] from the animal images. For analysis, the cats were placed into a group that best described their coat colour (Black, Black/White, Blue, Brown/Red/White Tabby, Brown Tabby, Calico, Dilute Calico, Grey Tabby, Red Tabby, Tortoiseshell (Tortie), White, White/Dark). 

#### 2.3.3. Objective Facial Measurements of Baby Schema 

The images were edited using GIMP-GNU Image Manipulation Program 2.0. The free select tool was used to draw around the cats’ faces to highlight the area required. The area selected was inverted and the region not selected was cropped out, leaving the cat’s face on a white background. The images were exported and saved in a PNG format with the background colour saved to maintain the blank background. The GIMP measuring tool, using pixels as units, was used to measure the cat images. 

**Figure 1:**
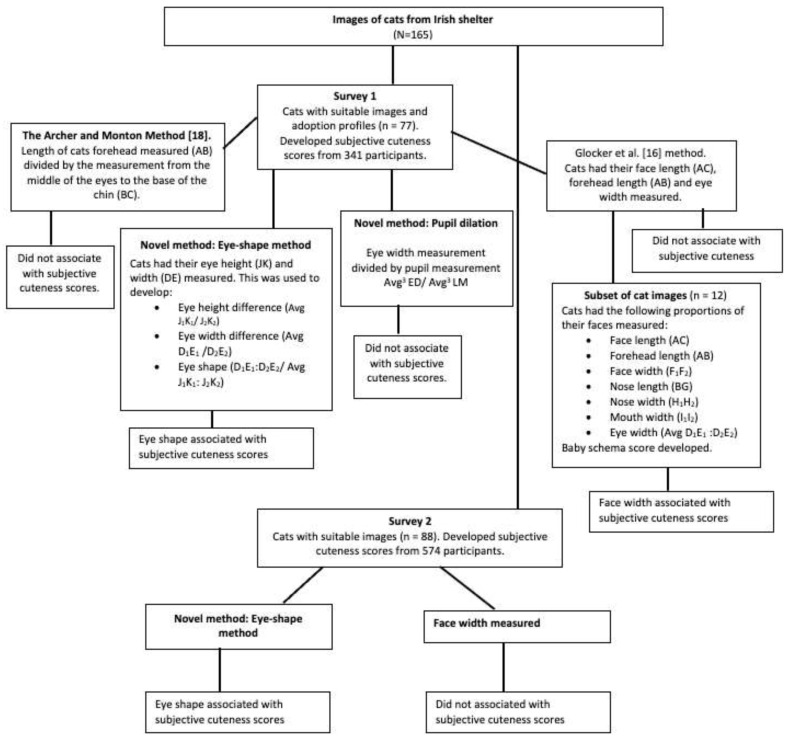
The path diagram gives an overview of the procedure followed during the development of the objective cuteness measures.

Within the literature, various methods have been used to measure facial features to create an objective measure of ‘cuteness’. The images used in survey 1 (n = 77) were used to explore the methods used by Archer and Monton [18] and Glocker et al. [16] that were previously found to be associated with subjective cuteness. Exploratory eye and pupil measurements were also carried out. After investigation using the images in survey 1, it was determined that the measurement for face width and the novel measurement for eye shape were potential subjective cuteness indicators. Therefore, the images used in survey 2 (images used in survey 1, plus the additional 88 images) had their face width and eye measurements taken to explore this further. One individual carried out each of the measurements three times per image with an average of each of these used for calculations. Please refer to the path diagram in Figure 1 for an overview of the objective cuteness measuring process which is explained in more detail below. To validate the measurements as objective measurements of cuteness, the measurements were compared to associated subjective cuteness scores (see Section 2.4.). 

##### Archer and Monton’s Facial Index Score

**Figure 2:**
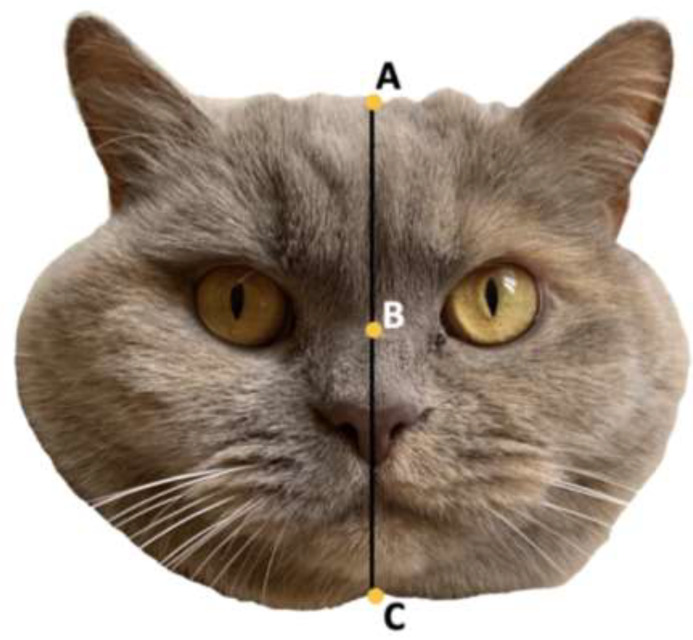
Archer and Monton (2011) Facial Index Score. Forehead length (AB) divided by middle of eyes to the chin (BC).

The facial index score created by Archer and Monton [18] is an objective rating of one of the characteristics of baby schema determined by Konrad Lorenz [31]. This score was created by taking the length of the forehead (AB) and dividing it by the measurement from the middle of the eyes to the base of the chin (BC), as seen in Figure 2. This score has been shown to be an indicator of baby schema in humans, animals, and teddy bears and to be associated with subjective ratings of cuteness [18]. The cats included in survey 1 had their face and forehead length measured to create an index score (n = 77).

##### Glocker Method

**Figure 3:**
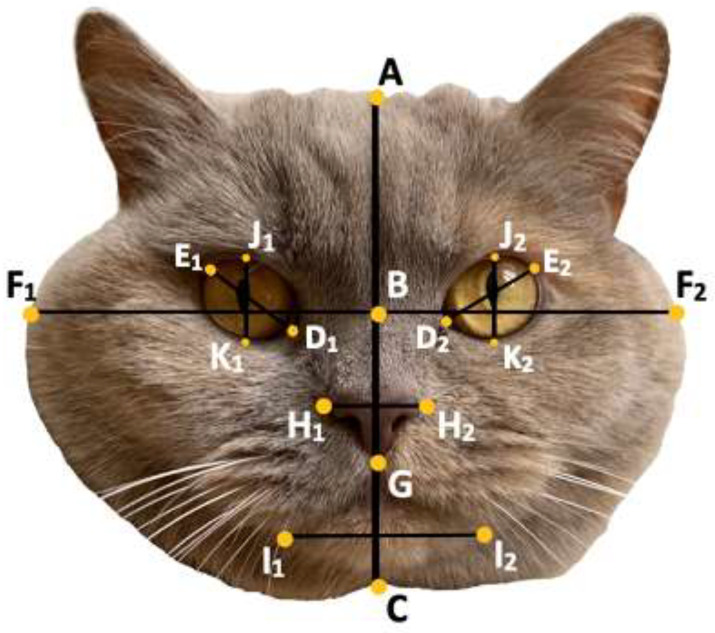
Glocker et al., (2009) Method. Face length (AC), forehead length (AB), face width (F_1_F_2_), nose length (BG) and width (H_1_H_2_), mouth width (I_1_I_2_), eye width (average of D_1_E_1_ and D_2_E_2_) and height (average of J_1_K_1_ and J_2_K_2_).

**Figure 4:**
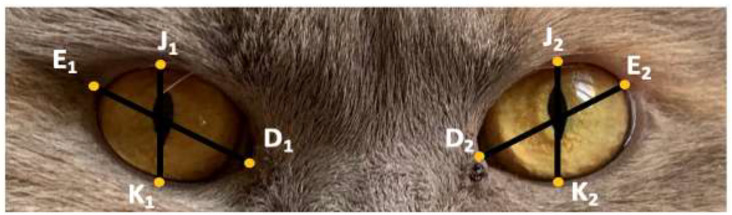
Novel eye-shape method. Eye height (JK; average of J_1_K_1_ & J_2_K_2_, eye width (DE; average of D_1_E_1_ & D_2_E_2_).

Glocker et al. [16] determined that high levels of the baby schema are expressed when the following facial proportions are high; face width, forehead length/face length, eye width/face width, and the following are low; nose length/head length, nose width/face width, mouth width/face width. To explore this, the cats (n = 77) included in the first survey had their face length (AC) and forehead length (AB) measured and their eye width (average of D_1_E_1_ and D_2_E_2_) measured, as shown in Figure 3. From these measurements, a subset of images was chosen to explore the remaining facial proportions that Glocker et al. [16] found to be important; face width, nose length and width, mouth width, and eye width. Three cats that had the highest and lowest forehead measurements and three cats with the highest and lowest measurements for eye width were chosen for preliminary analysis. Each of the 12 cats within this subset had their face length (AC), forehead length (AB), face width (F_1_F_2_), nose length (BG) and width (H_1_H_2_), mouth width (I_1_I_2_), and eye width (average of D_1_E_1_ and D_2_E_2_) measured, as shown in Figure 3 and Figure 4. The values of each facial parameter were converted to z-scores and a baby schema score was quantified as the mean of these scores. The additional cat images included in the second survey had their face width, along with their eye width and height (average of J_1_K_1_ and J_2_K_2_), measured. For additional information regarding measurement methodology, please see Glocker et al. [16].

##### Novel Method: Eye-Shape Method

Large eyes have associations with kawaii (cute Japanese culture) and happy, positive connotations, whilst small eyes are associated with untrustworthiness and negative emotions such as anger [32]. To assess if eye shape has associations with cuteness, each cat had its eye height (JK) and width (DE) measured (see Figure 4). For eye height and width, an average of both the left and right eye measurements were used. The eye measurements were used to create two dimensions; eye height difference (the difference between the measurement for the height of the left and right eye (J_1_K_1_–J_2_K_2_)) and eye width difference (the difference between the measurement for the width of the left and right eye (D_1_E_1_ and D_2_E_2_)), which explored the concepts regarding symmetry as an indicator of attractiveness [33]. A final dimension was developed which explored the shape of the eye (the difference between the average measurements for the height and width of the eyes (JK–DE)). The closer the eye shape dimension is to 1, the rounder the eye. Every cat within the study (n = 165) had the dimensions of its eyes measured.

##### Novel Method: Pupil Dilation

**Figure 5:**
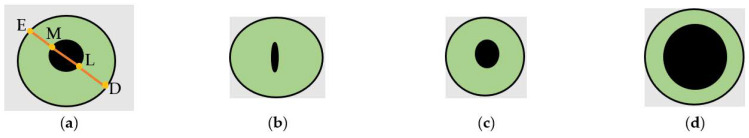
(**a**) Pupil dilation score. Eye width (DE) divided by pupil size (LM). (**b**) Example of small pupil. (**c**) Example of medium pupil. (**d**) Example of large pupil.

Large pupils along with large eyes are indicators of perceived cuteness in human infants [19], whilst previous research has shown that pupil size plays a role in communication and the building of trust [34,35], therefore, pupil size was explored. A pupil dilation score was created to investigate the potential effect this may have on perceived cuteness. The 77 cats which were included in survey 1 had the pupils (LM) and width (ED) of both eyes measured (LM), shown in Figure 5a. The average measurement for eye width (ED) was divided by the average of the pupil measurements to give an indication of pupil size. Scores of 1–2 were classed as large (Figure 5d), 2–7 were medium (Figure 5c) and 7–15 were small (Figure 5b). Of the 77 cats, 10 had large pupils, 44 had medium and 23 had small.

### 2.4. Subjective Measures of Baby Schema

Participants were recruited via social media (Facebook, Instagram, and Twitter) to provide subjective ‘cuteness’ ratings for each image. Prior to completing the survey, information regarding the study was given, including instructions and information about the cats shown. Participants gave their consent to the use of their data. There were two online surveys created; the first had the 77 cats which met all the criteria and had no missing information. The second survey included an additional 88 (resulting in 165 images in total) which were missing some information regarding home restrictions and social nature. Both surveys were created through Qualtrics and had the edited cat images uploaded and uniformed to 500 pixels in width. For the first survey, the participants were asked ‘How cute is this cat’ for each image and were given options on a Likert scale ranging from 1—‘Not Cute at all’ to 5—‘Extremely Cute’. The subjective cuteness scores for each cat image were developed from the mean cuteness score gathered from the participants. Participants were also asked, ‘Based on this image, would you say this cat had a positive (friendly) nature or a negative (unfriendly) nature?’ to identify whether perceived cuteness was related to perception of the cat’s social nature. As the additional cats included in the second survey did not have adoption profiles, the participants were only asked questions regarding perceived cuteness in this instance.

### 2.5. Statistical Analysis 

Statistical analysis was completed using SPSS version 26. Further analysis, which explored the demographic details gathered during both surveys, can be found in Jack et al., (in preparation). An overview of the participants’ demographic details can be found in Table S1 in the Supplementary Materials. 

#### 2.5.1. Cuteness Measurements 

To assess the relationships between subjective cuteness and the objective measurements, a series of correlation analyses were used. Spearman’s rank-order correlation was used to explore the relationship between subjective cuteness and the various objective measuring methods which were used on the first set of images from survey 1. Pearson’s product-moment correlation was used to ensure consistency between the subjective cuteness scores developed for the images in survey 1 that were included in survey 2. For the cat images included in the second survey (n = 165), the relationship between the novel eye shape and subjective cuteness score was established using Spearman’s rank-order correlation. A linear regression was run to further explore this relationship. 

#### 2.5.2. Length of Stay Analysis

To determine if there is an influence of subjective cuteness, factors found previously to affect the length of stay (cat age, sex, reason for relinquishment, coat colour) and other factors that are proposed to have an influence (adoption profile classification; positive or negative, homing requirements; singly or paired), a multiple regression with backward selection was run on the data collected in survey 1 (n = 77). It was determined that only subjective cuteness would be included in the regression model as exploratory analysis found that there was multicollinearity between the eye shape scores and subjective cuteness scores. As subjective cuteness is a more accurate reflection of perceived cuteness, considering the face as a whole, the eye shape score was removed from the model. A second multiple regression model was run on the data from survey 2 to determine if the length of stay was influenced by subjective cuteness ratings or other factors gathered at the shelter; age, sex, reason for relinquishment or coat colour (n = 165). 

## 3. Results

### 3.1. Facial Measurement Analysis

The relationship between subjective cuteness ratings and objective facial measurements can be seen in Table 1. Eye shape was the only measurement found to be statistically significant; there was a strong, negative association between eye shape and subjective cuteness ratings, *rs* (75) = −0.606, *p* < 0.001. 

For the preliminary analysis (n = 12), only one objective facial measure was found to be significant, with a statistically significant strong correlation between subjective cuteness and face width measurement, r(9) = 0.630, *p* = 0.038, seen in Table 2. The table shows the median and the range for the results of the analysis over the mean and the standard deviation due to the extremely low output figures for the baby schema score.

Analysis showed that the relationship between the subjective cuteness scores for surveys 1 and 2 to be linear with both sets of scores normally distributed, as assessed by Shapiro-Wilk’s test (*p* > 0.05), with no outliers. There was a statistically significant, strong positive association between the subjective cuteness scores from survey 1 and survey 2, *r*(75) = 0.925, *p* < 0.001.

For the images in survey 2 (n = 165), Spearman’s rank-order correlation found a statistically significant, strong negative correlation between subjective cuteness ratings and eye shape, *rs*(163) = −0.521, *p* < 0.001, indicating that the rounder the eye, the cuter the participant perceived the image to be. Linear regression was used to determine the effect of eye shape on participants’ subjective cuteness score. To assess linearity, visual inspection of a scatterplot of subjective cuteness scores against cat eye-shape scores indicated a linear relationship between the variables. There was homoscedasticity and normality in the residuals. There was one outlier with a subjective cuteness score of 2.96 and an eye shape score of ‘1’. This was deemed not to be extreme and was retained for analysis. 

The prediction equation was subjective cuteness = 4.119 + (−0.470 ∗ Eye shape). Eye shape statistically significantly predicted subjective cuteness, *F*(1, 97) = 42.538, *p* < 0.001, accounting for 20.7% of the variation in the cuteness scores with adjusted *R*^2^ = 20.2%, a small effect size.

### 3.2. Social Nature and Cuteness 

Participants’ perception of cat social nature was not included in the analysis due to the majority of images being rated as having a positive nature. 

### 3.3. Length of Stay Analysis

The backward selection multiple regression model run on the data collected in survey 1 found that only one variable, subjective cuteness rating, predicted length of stay, *F*(1, 75) = 6.530, *p* = 0.013, adj. *R*^2^ = 0.068. None of the factors which had been found previously to the affect length of stay (cat age, sex, reason for relinquishment, coat colour) or the factors which were proposed to have an influence (adoption profile classification; positive or negative, homing requirements; singly or paired), were retained (*p* > 0.05). The second multiple regression model which included all factors within survey 2; subjective cuteness ratings, age, gender, reason for relinquishment, coat colour; was not statistically significant, *F*(5, 159) = 1.045, *p* = 0.394, adj. *R*^2^ = 0.001. 

## 4. Discussion

### 4.1. Cuteness Measurement

The current study found that previous facial measurements that had been linked to baby schema and cuteness [16,18] were not associated with subjective cuteness scores. Forehead length had been found to be a key indicator of cuteness and baby schema in cats [18]. However, there appeared to be no association between subjective cuteness and this measurement within this study. This is potentially due to the disparity in the type of images used between the current study and previous studies. For example, Archer and Monton [18] used a small number of professional images; four images of adult cats; two with infant features and two without (one had to be manipulated to ensure it did not have infant features). These images had the cats facing straight on with bright professional lighting, while the images used in the current study were taken in a poorly lit shelter with most of the cats not fully facing forward. It is possible that Archer and Monton’s [18] facial index score is best while using professional images or when doing a comparative study to determine which image expresses the higher level of baby schema. The method developed by Glocker et al. [16] was also found not to associate with the subjective cuteness scores. While Glocker et al. [16] only used human infant faces, Borgi et al. [22] used this method successfully on cats. However, both studies used these measurements to manipulate images to express high and low levels of baby schema, therefore this method may be more suited to be used for comparison studies. 

Novel eye measurements (eye height difference, eye width difference, eye shape and pupil dilation) were explored to assess their importance in cuteness perception, as the size of the eye pupils is associated with a wide range of emotions [32,34,35]. Whilst eye height, width, or the difference between these was found not to be significant, eye shape (the difference between the average height and width) was moderately to strongly associated with cuteness. Despite the size of the eyes being one of the key elements of baby schema [11], it has been shown that, in human infants, eye size alone does not associate with perceived cuteness and that for it to be influential on cuteness perception, it should be paired with other facial elements [19,21,36]. Therefore, it could be assumed that this would be the same for cats and that while the size may be important, it will not be significant without taking into effect another measurement that is missing from the analysis. As face width has been shown to potentially be an indicator of cuteness in cats and rounder faces in human infants are considered cuter [36], using this measurement along with eye measurements to develop a score that reflects these could be a more valid indicator. The novel measurement which reflected the shape of the eye was found to be statistically significant. There was a negative association between subjective cuteness scores and the eye shape score, indicating that as the subjective cuteness score increases, the eye shape score decreases. Furthermore, it has been found that the closer the eye shape score is to 1 (the rounder the eyes), the higher the subjective cuteness. This is unsurprising considering that artists trying to convey cuteness in their illustrations have a tendency to draw their characters with round large eyes; this can be seen in the evolution of the Disney character Mickey Mouse, who has evolved rounder and softer eyes in an effort to appear more innocent and cute [37]. It was expected that pupil size would have a strong influence on cuteness perception due to young infants having larger pupils [38,39] with illustrators using enlarged pupils to make their characters appear cuter [37]. The lack of association could be due to the quality of the images, the minimal number of images with small pupils (n = 10) or perhaps pupil size is only relevant when eye size is taken into consideration. Further studies could use the same images with manipulated pupil sizes to assess their potential influence on cuteness perception whilst ensuring there are no other confounding factors. There are several advantages of using the novel eye shape method developed through this study over the previously explored approaches [16,18,22,23]. Eye shape scoring requires minimal knowledge of advanced imaging software and does not require professional images, allowing images taken from real-life situations to be analysed. This is important as the other approaches appeared not to work with this type of image. In addition, to create the eye shape score, only the eyes need to be measured and a very simple calculation is required, unlike the methods employed in other studies [16,22].

### 4.2. Length of Stay

Despite numerous studies citing phenotypic traits (coat colour, sex, and age) and environmental circumstances (relinquishment reasons) as major influential factors on adoption speed in shelters [4,40,41], none of these factors were found to be statistically significant in the current study. There was a larger portion of female cats in the study than males and they do appear to take longer to be adopted (see Table S2 in the Supplementary Material). However, sex was not found to be related to the length of stay. Whilst it is difficult to determine why the study found conflicting results to the previous research, it is possible that as this is an Irish shelter, there may have been cultural differences in terms of preference for coat colour or sex, et cetera. However, the exact reason remains uncertain. As this is data from only one shelter, future studies could gather information from several shelters, which might give a better picture.

Neither of the statistical models provided an understanding of the factors that affect cats’ LoS in the shelter environment. Therefore, the hypothesis regarding ‘cute’ cats having a shorter LoS than less ‘cute’ cats is rejected. It could be that these factors are not as important as they have been suggested to be in the adoption of cats. As with the adoption of human infants, physical appearance has been regularly cited as one of the most important factors in kitten adoption, while behaviour was cited as being most important when adopting an adult cat [24,42,43]. Several studies have found that active cats, who exhibit playful behaviours, and those that interact with potential adoptees, have greater chances of being adopted [5,40,42,43,44,45]. Brown and Morgan [4] suggested that behaviour may play a key role in adoption and suggest that male cats may be adopted quicker due to their temperament. This should be explored in future research. Brown and Morgan [4] observed that the male cats seemed to exhibit more playful behaviours, appeared to be less reserved and would initiate contact with potential adopters more readily than females. However, Bennett et al. [46] found that there was little difference in personality between the sexes, with only a slight difference in ‘demanding’ behaviours between neutered females and non-castrated male cats. Instead, cat temperament has been found to be influenced by the temperament of their fathers and by their early socialisation [47,48], with ‘friendly’ fathers producing ‘friendly’ offspring. 

### 4.3. Adoption Profiles and Paired-Homing Requirements 

For dogs, the use of certain language when advertising for adoption has been found to be important in the reduction of LoS [49]. Contrary to our hypothesis and the effect it has on dog adoption, there was no effect of the use of language in the adoption profiles. Therefore, this hypothesis is rejected, the wording of cat adoption profiles may consequently be less influential on adoption decisions than dog adoption profiles. 

There was no effect of the paired-homing requirements on the length of stay. However, only 21 out of 77 cats had this requirement. When looking at the breakdown of this requirement (Table S3 of the Supplementary Material), cats that were required to be homed with another cat did on average take longer to rehome. As this was not statistically significant, the hypothesis that cats with paired-homing requirements will take longer to adopt than those without paired-homing requirements is rejected. To the knowledge of the researchers, no other studies have addressed the effect that paired-homing requirements could have on LoS. 

### 4.4. Social Nature and Cuteness 

The majority of the cat images were rated overwhelmingly as positive, resulting in the participant’s perception of the cat’s social nature not being included in the analysis. The hypothesis that cats rated as ‘cuter’ will be perceived as having a positive nature compared to those with lower cuteness ratings could not be investigated, therefore this hypothesis cannot be rejected nor accepted. 

### 4.5. Limitations and Future Directions

There were a few limitations within the study, particularly regarding the images used. In dog adoption profiles, the images were found to have an effect on LoS, with those with higher quality images and positioning of the dog influencing adoption speed [50]. The images used in the study were reflective of the type of image used in adoption profiles in this shelter. Given the real-world context, it is possible that some of the cat faces in the images were off-centre. However, only the best images were used from the original image database of 1281 cats. At the same time, lowering the sample size available for analysis ensured that the images were as suitable as possible. Had all of the cats’ faces been in the correct position, then the association with the subjective cuteness scores could have been stronger, but as the data and images were collected retrospectively from the cat shelter, this was not possible for this study. Ideally, instead of using images from adoption profiles that have been taken by shelter staff, taking images within a shelter would be preferential. Future studies could analyse the quality of the image and the positioning of the animal versus LoS. Each of the images was edited to leave only the face of the cat, and to ensure that there was no influence from either the background or the body of the cat on scoring. This proved to be quite difficult, particularly with the poorly lit images, causing difficulties in identifying the outline of the face and positioning of the chin. Therefore, not all the images may be a perfect representation of the cat in them. Future research could explore the effect of using more than one image to represent each individual cat.

Behaviour has been shown to be an important factor in adoption [5,6,10]. Therefore, not including behaviour as a factor is a study limitation. As this study used retrospective data, the only way to include behaviour was to use the adoption profiles as a proxy. Future research should assess how behaviour and baby schema levels affect LoS in shelter cats. This could be assessed by questioning shelter staff, assessing the behaviour through observation or by temperance testing. Eye shape measurements should be included in any additional studies to further validate their reliability as an accurate measurement of cuteness in cats. The lack of information regarding the cat’s health records could also be regarded as a limitation. Although this puts the study in line with others [4,7,51], it does not give a completely accurate portrayal of LoS for some female cats, who could be pregnant or nursing, which could be skewing the data towards male cats having a shorter LoS. Any additional studies looking to further explore LoS or the role that cuteness may play in adoption should ensure they have the health records or information regarding pregnancy and/or nursing.

The current study found that adoption profiles did not influence the cat’s LoS. However, there was a difference between the average and minimum LoS. As the adoption profiles were categorised based on the language used and participants did not read the profiles, it would be beneficial for future studies to assess positive and negative adoption profiles by investigating the level of interest these profiles generate. Future studies could utilise an experimental design in which the same image is shown along with different blurbs to help elucidate the exact effect of adoption profiles on willingness to adopt. However, should the use of positive language increase willingness to adopt, it would not be recommended to use this technique on cats that require additional behavioural support as the adopter would be ill-prepared to provide the appropriate requirements for the cat and would have very different expectations as to how they would expect the cat to behave. Ensuring cats and their adopters are well suited should be a priority and that adopters have been well educated to promote animal welfare and ensure retention [52].

Lastly, the lack of variation in subjective cuteness scores, and the majority of the participants rating the cats as having a positive nature, could reflect the fact that those that chose to participate in this research may have a particular interest in cats. Using paid participants may have given a more accurate reflection of how cats are perceived by the general population with less bias. However, as this study looked to determine if cuteness has an influence on the cats’ LoS in an animal shelter, the potential bias of cat lovers would have created an accurate reflection of the association of cuteness to LoS as it would be those with a preference for cats who would be seeking to rehome them.

## 5. Conclusions

It is not conclusive if baby schema, or ‘cuteness’, plays a role in the adoption of cats from shelters. Further research is needed to assess the effect of ‘cuteness’ on LoS by assessing this in a larger number of cats across several animal shelters. Future studies should also consider including the effect that cat behaviour and health have on adoption selection. Eye shape as an indicator of cuteness in cats should be explored further as it is simple to measure relative to other baby schema measurements and appears to be associated with cuteness regardless of the quality of the image. This measure needed to be further validated. However, it provides a less time-consuming and simpler method of objective measurement of infant features. This will provide future researchers with the tools to identify the level of ‘cuteness’ or baby schema in a cat image much easier for further research. 

If cuteness is an important factor for rehoming, it is suggested that shelters use images in adoption profiles that enhance the cat’s cuteness, with the cats’ eyes open wide to attract the attention of potential adopters. With such high numbers of cats housed within cat shelters, it is imperative we understand adoption selection as much as possible to utilise the tools at our disposal in order to reduce LoS and improve cat welfare. 

## Figures and Tables

**Table 1:** Relationship between subjective cuteness ratings and objective facial measurements from images (n = 77) in survey 1 using Spearman’s rank-order correlation.

	Archer and Monton [18]		Glocker et al. [16]			Novel Method			
	Facial Index	Forehead Prop ^1^	Face Width	Eye Width	Eye Height	Diff ^2^ Eye Height	Diff ^2^ Eye Width	Eye Shape	Pupil Size
	AB/BC	AB/AC	F_1_F_2_	Avg ^3^ D_1_E_1_:D_2_E_2_	Avg ^3^ J_1_K_1_: J_2_K_2_	Avg ^3^ J_1_K_1_/J_2_K_2_	Avg ^3^ D_1_E_1_/D_2_E_2_	Avg ^3^ D_1_E_1_:D_2_E_2_/Avg ^3^ J_1_K_1_: J_2_K_2_	Avg ^3^ ED/Avg ^3^ LM
Mean	0.860	0.460	1189.57	209.05	147.85	1.00	1.00	1.43	5.346
SD	0.156	0.046	525.16	92.18	66.26	0.089	0.072	0.217	3.71
Correlation Coefficient	−0.013	−0.003	−0.012	−0.041	0.123	0.073	0.033	−0.606 **	−0.073
*p*-value	0.911	0.981	0.857	0.720	0.287	0.529	0.777	<0.001	0.528

^1^^.^ Proportion. ^2.^ Difference between. ^3.^ Average **^.^ Correlation is significant at the 0.01 level (2-tailed).

**Table 2:** Results from Spearman’s rank-order correlation of subjective cuteness ratings and subset of images (n = 12), measured using the Glocker et al. [16] method.

	Face Length AC	Forehead Length AB	Face Width F_1_F_2_	Nose Length BG	Nose Width H_1_H_2_	Mouth Width I_1_I_2_	Eye Width Avg ^1^ D_1_E_1_:D_2_E_2_	Baby Schema Score
Median	1110	0.498	1282.33	0.303	0.169	0.293	0.183	0.010
Range	1764.33	0.180	1792.67	0.060	0.080	0.140	0.070	1.17
Correlation Coefficient	0.558	−0.170	0.630 *	−0.235	0.162	−0.239	−0.251	0.110
*p* value	0.074	0.618	0.038	0.486	0.635	0.480	0.456	0.748

*. Correlation is significant at the 0.05 level (2-tailed). ^1.^ Average.

## Data Availability

The data presented in this study are available on request from the corresponding author. The data are not publicly available as the data was acquired from a third-party source.

## Supplementary Materials

The following supporting information can be downloaded at: https://www.mdpi.com/article/10.3390/ani12111461/s1, Table S1. Demographic details of participants that took part in the subjective cuteness rating in survey 1 (n = 341) and survey 2 (n = 574); Table S2. Days to adoption for cats in shelter separated by sex for both surveys; Table S3. Details of LoS for cats in survey 1, with a breakdown for adoption profiles and homing requirements
