# Supplementary material for: The Effect of Baby Schema in Cats on Length of Stay in an Irish Animal Shelter
Source: Animals (Basel). 2022 Jun 4;12(11):1461. doi: 10.3390/ani12111461 (PMC9179851; doi:10.3390/ani12111461)
Supplement: Supplementary file 1 [file animals-12-01461-s001.zip › animals-1703417-supplementary.pdf]

animals MDPI

Supplementary Material

Table S1. Demographic details of participants that took part in the subjective cuteness rating in survey 1 (n = 341) and survey 2 (n = 574)

**Demographic Information**	**Percentage**	
**Gender**	**Survey 1**	**Survey 2**
Male	15.8	22
Female	82.1	73.9
Self-Identify	2.1	4.2
**Age**		
18-24	8.8	5.4
25-39	60.1	54
40-59	28.2	34.7
60+	2.9	5.9

Table S2. Days to adoption for cats in shelter separated by sex for both surveys.

**Survey 1**						
	N	Min	Max	Mean	Median	Std. Dev
Male	23	18	475	104	71	125.37
Female	54	15	453	108	78.50	85.10
Survey 2						
Male	58	13	505	87	51	105.36
Female	107	1	453	100	73	87.192

Table S3. Details of LoS for cats in survey 1, with a breakdown for adoption profiles and homing requirements.

	**Average LoS**	**Min**	**Max**	**N**
**Adoption Profile**				
Positive	**100**	**15**	**475**	**47**
Negative	**118**	**28**	**475**	**30**
**Homing requirement**				
Single	**101**	**15**	**453**	**56**
Paired	**125**	**21**	**475**	**21**
